# Anti-Programmed Cell Death-1 Antibody and Dasatinib Combination Therapy Exhibits Efficacy in Metastatic Colorectal Cancer Mouse Models

**DOI:** 10.3390/cancers14246146

**Published:** 2022-12-13

**Authors:** Hiroki Kadota, Ryo Yuge, Daisuke Shimizu, Ryo Miyamoto, Rina Otani, Yuichi Hiyama, Hidehiko Takigawa, Ryohei Hayashi, Yuji Urabe, Yasuhiko Kitadai, Shiro Oka, Shinji Tanaka

**Affiliations:** 1 Department of Gastroenterology, Hiroshima University Hospital, Hiroshima 734-8551, Japan; 2 Clinical Research Center in Hiroshima, Hiroshima University Hospital, Hiroshima 734-8551, Japan; 3 Department of Endoscopy, Hiroshima University Hospital, Hiroshima 734-8551, Japan; 4 Department of Gastroenterointestinal Endoscopy and Medicine, Hiroshima University Hospital, Hiroshima 734-8551, Japan; 5 Department of Health Sciences, Prefectural University of Hiroshima, Hiroshima 734-8558, Japan

**Keywords:** cancer-associated fibroblasts, metastatic colorectal cancer, platelet-derived growth factor receptor, dasatinib, programmed cell death-1 antibody

## Abstract

**Simple Summary:**

Cancer-associated fibroblasts (CAFs) are abundant in colorectal cancer (CRC) tissues and are essential for tumor growth. Histological analysis of CRC clinical specimens showed that the number of CAFs in liver metastases correlated with the number of CAFs in primary tumors. We hypothesized that the presence of CAFs in metastatic CRC contributes to immune evasion, and thus evaluated the efficacy of dasatinib in targeting CAFs in combination with immunotherapy in a mouse model of liver metastasis. Immunotherapy in combination with dasatinib was shown to reduce stromal components and subsequent immune cell infiltration and promote the activation of tumor immunity and tumor regression in liver metastatic tumors with abundant CAF components. These results suggest that CAFs play an important role in the immune evasion of CRC and that dasatinib is a promising combination agent for increasing immunotherapy sensitivity in metastatic CRC.

**Abstract:**

In this study, we investigated the in vivo metastasis suppression effects of the platelet-derived growth factor receptor inhibitor dasatinib, which targets cancer-associated fibroblasts (CAFs), in combination with an anti-programmed cell death-1 (PD-1) antibody. We classified clinical CRC cases as inflamed, excluded, or desert using immunohistochemical analysis and evaluated the tumor stroma. The excluded type was the most common, and cases with high-volume stroma in the primary lesions also had a high stromal volume in the liver metastatic lesions. Liver-metastasis mouse models with different stromal volumes were established and treatment-induced changes in the tumor immune microenvironment were evaluated. The anti-PD-1 antibody alone exhibited a therapeutic effect for the liver metastases with low stromal volumes but not for the liver metastases with high stromal volumes. In contrast, antitumor effects were observed with anti-PD-1 antibody/dasatinib combination therapy even in the liver metastases with high stromal volumes. Combination therapy reduced the stromal volume, promoted immune cell infiltration, induced antitumor cytotoxic T-cell responses, activated antitumor immunity, and promoted tumor regression. These results suggest that CAFs play an important role in the immune evasion of CRC and that anti-PD-1 antibody/dasatinib combination therapy has potential as a treatment option for patients with metastatic CRC for whom immunotherapy alone is ineffective.

## 1. Introduction

Immune checkpoint inhibitors, including anti-programmed cell death-1 (PD-1) antibodies, have recently shown excellent results in clinical trials, thereby increasing their potential for use [1,2,3,4,5,6,7]. The efficacy of immune checkpoint inhibitors against colorectal cancer (CRC) is limited, and the prognosis for metastatic CRC remains poor, with one-third of CRC-related deaths attributed to liver metastasis. There is a clear need to elucidate the mechanisms that affect the immune responses in CRC tumors. Hence, the evaluation of combination therapies that enhance tumor immunity has become a major point of interest in the field of cancer biology.

Tumor tissues consist of both cancer and stromal cells, including various immune cells. The interaction between the cancer and stromal cells is important for the growth and progression of cancer. Fibrous stromata are known to develop in CRCs around the cancer foci via a process called “stroma-genesis” or “stromal reaction” [8,9]. The main constituents of fibrous stroma are cancer-associated fibroblasts (CAFs). Interactions between CAFs and cancer cells promote tumor development and progression [8]. We have previously shown that platelet-derived growth factor receptor (PDGFR) is highly expressed in the CAFs present in the stroma of CRC [10]. Furthermore, the administration of a dasatinib strongly suppressed the stromal response and produced a therapeutic effect in a mouse model of orthotopic transplanted tumors [11,12].

We previously considered whether CAFs, which are abundant in CRC tumor tissues, act as treatment barriers and limit immune cell infiltration [13]. We found that a combination treatment approach with CAF-targeted drugs and immunotherapy can be effective in a mouse model of CRC that uses the orthotopic transplantation of tumors [13]. However, the efficacy of this combination therapy in metastatic CRC has not yet been determined.

The metastasis of cancer cells generally involves decreased cell–cell adhesion, increased motility and mobility, invasion into the blood and/or lymphatic vessels, transport through the circulatory system, extravasation, and eventual dissemination to remote areas [14]. Metastasized cancer cells pass through the circulatory system not only as single cells but also as groups of cells containing CAFs that accompany the cancer cells to distant metastatic foci [15,16]. However, there are currently no reports regarding the pathological findings that support the above knowledge or the relationship between CAFs and tumor immunity. We hypothesized that the combined use of CAF-targeted drugs and immunotherapy could be a rational therapeutic strategy for metastatic CRC if the CAFs that were abundant in the primary foci co-metastasized along with the cancer cells to the liver metastasis foci.

In recent years, three histological phenotypes have been proposed for the classification of tumor immunity: inflamed, in which immune cells infiltrate the cancer focus; excluded, in which immune cells accumulate in the stroma but fail to infiltrate inside the cancer focus; and desert, in which no immune cells are found in the tumor immune microenvironment (TIME) [17,18]. However, there are currently no reports regarding the differences in the stromal amounts between these phenotypes.

In the current study, we used clinical CRC specimens to investigate the proportion of CAF components in the primary and liver metastatic foci according to immune tissue phenotypes. We also established an experimental liver metastasis mouse model that reproduced the immunohistochemical phenotypes and employed this model to conduct therapeutic studies using an anti-PD-1 antibody and a dasatinib. The objective of the in vivo studies was to determine the efficacy of the promising candidate dasatinib as a combination drug to increase the sensitivity of cancer immunotherapy for the treatment of metastatic CRC.

## 2. Materials and Methods

### 2.1. Human CRC Clinical Specimens

Surgically resected primary and liver metastatic specimens were collected from 165 CRC patients. The study participants were patients diagnosed with CRC who had undergone surgical resection at Hiroshima University (Hiroshima, Japan) between 2013 and 2019. The study was conducted in accordance with the Declaration of Helsinki and approved by the Hiroshima University Hospital Facility Review Committee (approval E-1237). Because anonymized data were used, the institutional review board waived the requirement for each patient to provide informed consent.

### 2.2. Reagents

Mouse colon cancer-derived CT26 cells were obtained from the American Type Culture Collection (Manassas, VA, USA). InVivoMAb anti-mouse PD-1 antibody (CD279; Bio X Cell, Lebanon, NH, USA) and dasatinib (Tokyo Chemical Industry, Tokyo, Japan) were used for the therapeutic studies. The primary antibodies used are detailed in the Supplementary Materials and Methods (File S1). TruStain FcX Plus antibody (anti-mouse CD16/32; BioLegend) was used for blocking, and 4′,6-diamidino-2-phenylindole (Dojindo Laboratories, Kumamoto, Japan) was used to stain the dead cells. An RNeasy Mini Kit was used for RNA extraction (Qiagen, Hilden, Germany).

### 2.3. Evaluation of Human CRC Stromal Amounts in Primary and Liver-Metastasis Foci

Formalin-fixed, paraffin-embedded tissues prepared from resected tumor specimens were serially sectioned at 4-µm thickness. Tissue sections were immunostained using anti-CD8 and anti-α-SMA antibodies as previously described [19]. CD8 immunostaining was used to classify the TIME of human CRC specimens into immune-tissue phenotypes of inflamed, excluded, or desert types, as detailed in the Supplementary Material. Clinicopathological features of patients classified according to the immune-tissue phenotype of their resected specimens were compared. Immunostaining for α-SMA was used to evaluate stromal amounts in human CRC primary and liver metastasis foci, as described in the Supplementary Material.

### 2.4. Animals

Female BALB/c mice (9 weeks old) were obtained from Charles River Laboratories (Tokyo, Japan) and housed under specific pathogen-free conditions. The experiments were approved by the Animal Experiment Committee of Hiroshima University (approval A20-35). All experiments were conducted in accordance with the Animal Research: Reporting of In Vivo Experiments guidelines and the U.K. Animals (Scientific Procedures) Act of 1986 and EU Directive 2010/63/EU on animal experiments.

### 2.5. Cell Culture and CAF Preparation

CT26 cells were maintained in Dulbecco’s modified Eagle’s medium (DMEM) supplemented with 10% fetal bovine serum and a penicillin–streptomycin mixture. CT26 cells were injected subcutaneously into BALB/c mice to induce tumors and generate CAFs, as detailed in the Supplementary Material. The isolated cells were sorted using fluorescence-activated cell sorting (FACS) and CD45^−^EpCAM^−^PDGFR^+^ cells were used as CAFs.

### 2.6. Cancer Cell Proliferation Assays

The ability of dasatinib and the anti-PD-1 antibody to suppress the growth of cancer cells and fibroblasts was investigated using an IncuCyte time-lapse assay system (Essen BioScience, Ann Arbor, MI, USA). CT26 cells or CAFs were seeded into 24-well plates at a density of 6 × 10^4^ cells/well and cultured with dasatinib (10, 100, 500 µM, or 1 nM) or anti-PD-1 antibodies (1, 5, or 10 nM). The confluency percentage was measured every two hours for seven days.

### 2.7. CRC Liver-Metastasis Mouse Models

Inflamed- and excluded-type CRC liver metastasis mouse models were established as described in the Supplementary Material. Briefly, for the inflamed-type model, CT26 cells were injected into the spleens of female BALB/c mice to create liver metastatic tumors with few stromal components. For the excluded-type model, CT26 cells and CAFs were injected into the mice to create liver metastatic tumors rich in stromal components.

### 2.8. Therapeutic Experiments Using CRC Liver-Metastasis Mouse Models

Anti-PD-1 antibody treatment efficacy was evaluated using the inflamed-type CRC liver metastasis model, as detailed in the Supplementary Material. Animals were divided into treatment and control groups. The anti-PD-1 antibody treatment group received a total of three treatments. The control group was treated with isotype control IgG antibody. Tumors were imaged in vivo using a Lumina II system, and the mice were sacrificed 14 days post-transplantation. The efficacy of the combination treatment was evaluated using an excluded-type CRC liver metastasis model. Animals were divided into four treatment groups: anti-PD-1-antibody treatment, dasatinib treatment, combined treatment, and control. The anti-PD-1-antibody treatment group received three treatments. The dasatinib treatment group received daily oral administration of dasatinib on days 1–13 after transplantation. The combined treatment group received both the three anti-PD-1 antibody treatments and the daily dasatinib treatment. The control group received three isotype control IgG treatments, along with daily oral administration of isotype control IgG. Tumors were imaged in vivo using a Lumina II system. Mice were monitored until death from natural causes or until sacrifice on day 14 post-transplantation. All tissue specimens were collected during necropsy, as described in the Supplementary Material.

### 2.9. RNA-sequencing (RNA-seq) Analysis and Gene Set Enrichment Analysis (GSEA)

Excluded-type liver metastatic tumors from mice treated with anti-PD-1 antibodies as monotherapy or in combination with dasatinib therapy were dissected on day 14 post-transplantation. Liver metastatic tumors were prepared for RNA-seq and processed as detailed in the Supplementary Material. Samples were analyzed using GSEA to determine the differential modulation of molecular pathways.

### 2.10. FACS Analysis of Immune-Cell Surface Antigen Expression

Necropsies were performed on day 14 post-transplantation on mice treated with anti-PD-1 antibodies alone or in combination with dasatinib. Liver metastatic tumors were removed and processed for FACS analysis, as detailed in the Supplementary Material.

### 2.11. Statistical Analysis

Clinicopathological features were analyzed using the χ^2^ test or Fisher’s exact test to compare categorical data, and a Student’s *t*-test or Wilcoxon rank-sum test was used to compare continuous data. Spearman’s rank correlation coefficient was used to index correlations. A log-rank test was used to compare the Kaplan–Meier curves and to analyze overall survival. Statistical significance was set at *p* < 0.05. All statistical analyses were conducted using R version 4.0.3 (http://www.r-project.org, accessed on 10 October 2020).

## 3. Results

### 3.1. Stromal Amounts in Human CRC According to Immune-Tissue Phenotype

**Figure 1:**
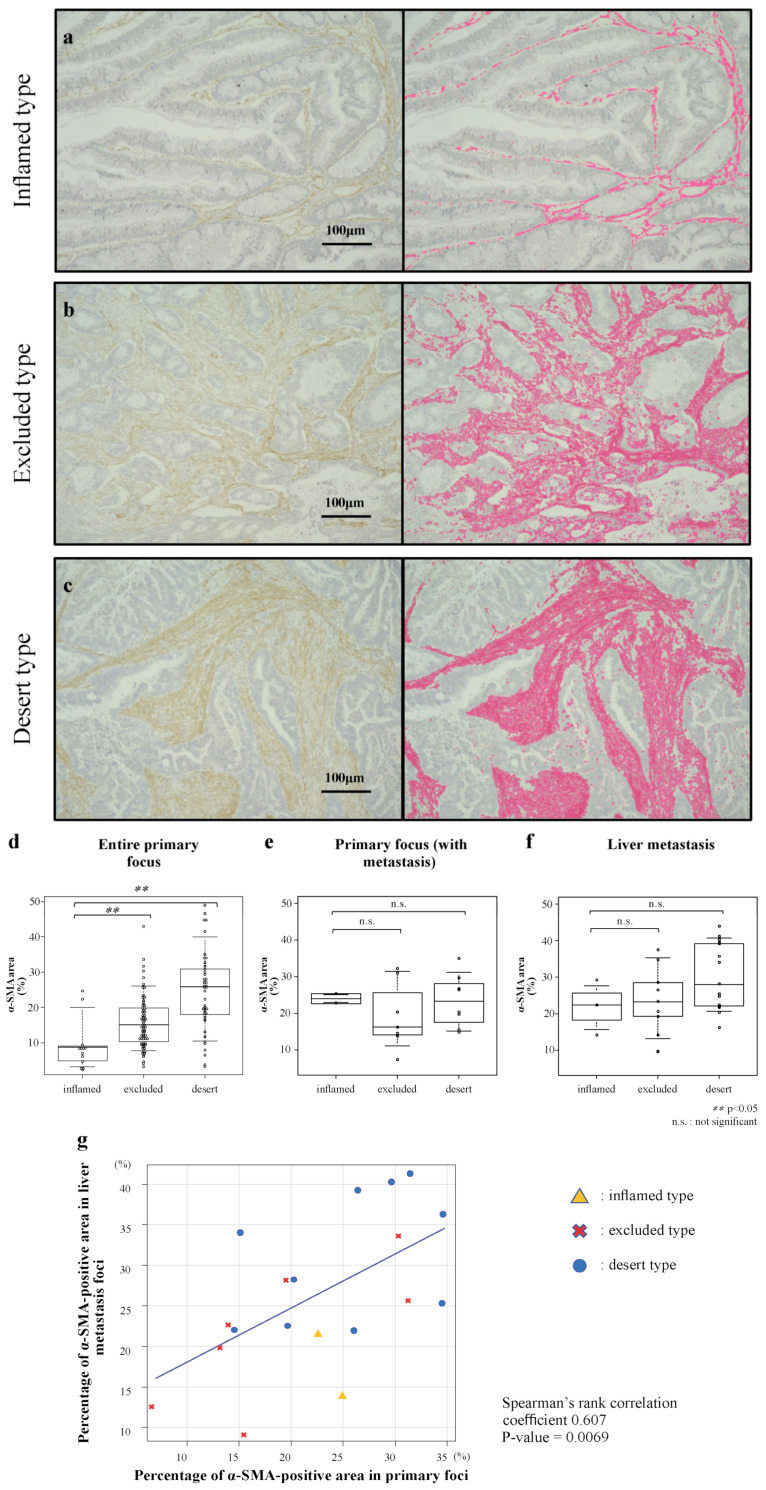
Analysis image of CAF-involved areas in human CRC for each phenotype. The left side shows immunostained images of α-SMA of human CRC primary foci, while the right side shows the images analyzed using BZ-X analysis software. The areas that were positive by immunostaining were automatically identified and shown in pink. The interstitial areas of (**a**) inflamed type, (**b**) excluded type, and (**c**) desert type are shown. The magnification of the micrographs is 200×. The size bar represents 100 μm. Percentage of CAFs in CRC primary foci and liver metastases determined based on phenotype. Quantified percentage of α-SMA-positive areas in (**d**) the entire CRC primary foci, (**e**) the primary foci in liver-metastasis cases, and (**f**) all liver metastases. (**g**) Scatterplot demonstrating the correlation between α-SMA-positive areas of primary foci and liver metastases in liver-metastasis cases.

First, we classified the surgically resected human CRC specimens into immune-tissue phenotypes of inflamed, excluded, or desert types using CD8 immunostaining (Supplementary Figure S1). Based on the phenotype classification, the excluded-type cases were the most common (54.5%), whereas the inflamed-type cases were the least common (10.3%). No association was found between the phenotype classification and other clinicopathological characteristics, including sex, age, tumor localization, tumor stage, vascular invasion, and histological type (Supplementary Table S1). Next, the area of positive immunostaining for the CAF marker α-SMA in the primary foci was quantified to validate the relationship between the phenotype and CAF prevalence in the primary CRC foci (Figure 1). Quantification of the CAF-involved areas in the primary foci and phenotype comparisons revealed that the area with the CAFs was significantly higher in the excluded and desert types than in the inflamed type (*p* < 0.05%; Figure 1d).

### 3.2. Correlation of Stromal Amounts in Primary Foci and Liver Metastatic Foci of Human CRC

We investigated whether there was a correlation between the CAF prevalence in the primary and liver metastasis foci by immunostaining for α-SMA in the specimens of the liver metastasis foci that were surgically resected and for which phenotype classification was completed. The analysis of the CAF-involved areas of the primary foci in the cases with liver metastasis revealed no significant differences among the phenotypes (Figure 1e) or within each phenotype of the liver metastasis foci (Figure 1f). A scatter plot of the liver metastasis cases was generated to investigate the correlation between the CAF prevalence in the primary foci and that in the liver metastasis foci. The results showed a correlation between the two foci types with a correlation coefficient of 0.61 (Figure 1g). Interestingly, the two cases that were high outliers in the inflamed type, with high levels of α-SMA-positive areas, were the liver metastasis cases (yellow triangles in Figure 1g). These results demonstrate that the cases with abundant CAFs in the primary foci also exhibited abundant CAFs in the liver metastasis foci, regardless of the phenotype.

### 3.3. Effects of Anti-PD-1 Antibody and Dasatinib Treatment on the Proliferative Potential of CRC Cells and CAFs

We investigated the effects of the anti-PD-1 antibody and dasatinib treatment on the proliferative potential of the CT26 cells and CAFs in vitro. The proliferation of the CAFs was suppressed in a concentration-dependent manner when the dasatinib was administered (Supplementary Figure S2A). In contrast, the proliferation of the CT26 cells was not suppressed, even when the dasatinib was administered at the same concentration that affected the CAFs (Supplementary Figure S2B). The anti-PD-1 antibody treatment did not affect the proliferative ability of the CAFs or CT26 cells at any of the various concentrations evaluated.

### 3.4. Effects of Immunotherapy on Inflamed-Type CRC Liver Metastasis

**Figure 2:**
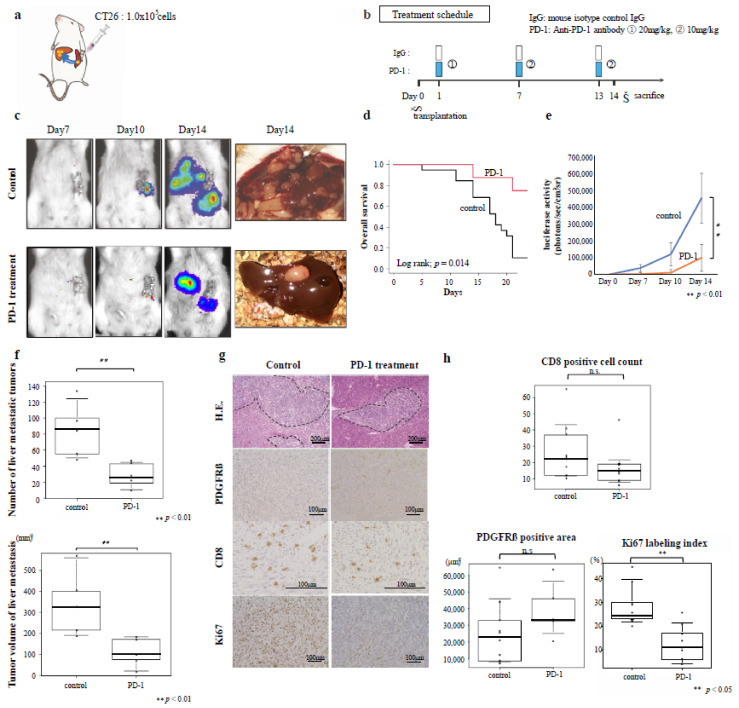
Anti-PD-1 antibody treatment and inflamed-type colon cancer liver metastasis. The inhibitory effect of anti-PD-1 antibody on liver metastasis was evaluated using a mouse model of inflamed-type colon cancer. (**a**) Tumor transplantation design and transplanted cell content. (**b**) Treatment schedule. Inflamed control, n = 8; Inflamed PD-1, n = 8. (**c**) Changes in tumors over time as observed by luminescence imaging (Days 7, 10, and 14 post-transplantation) and gross observation of the tumors with the naked eye after sacrifice on day 14 post-transplantation. (**d**) Kaplan–Meier survival curve. Control, n = 19; PD-1, n = 8. (**e**) Quantification of changes over time according to luciferase activity. (**f**) Tumor number and volume of metastasized tumors. (**g**) Representative micrographs of hematoxylin and eosin (HE)-stained (100× magnification) and immunostained (200× magnification) tumors. Immunostaining was performed using PDGRβ-, CD8-, and Ki-67-specific antibodies. (**h**) Quantification of the number of CD8-positive cells, PDGFRβ-positive area, and Ki67-positive cell percentage of metastatic tumors. n.s. = not significant.

The therapeutic effect of immunotherapy on inflamed-type CRC liver metastasis was investigated using a liver metastasis mouse model in which only CT26 cells were transplanted into the spleens of mice (Figure 2a), followed by treatment with an anti-PD-1 antibody (Figure 2b). A representative image of a liver removed 14 days after transplantation is shown in Figure 2c (right panel). Overall survival was significantly improved in the anti-PD-1 antibody treatment group compared with that of the control group (*p* = 0.014; Figure 2d). The control group tended to lose more body weight than the anti-PD-1 antibody group (Supplementary Figure S3A). Luciferase activity was significantly lower in the anti-PD-1 antibody treatment group than in the control group at 14 days post-transplantation (*p* < 0.01; Figure 2e). The number and volume of liver metastatic tumors were both significantly lower in the anti-PD-1 antibody treatment group than in the control group (*p* < 0.01; Figure 2f). When only CT26 cells were transplanted into the spleen, the expression of the CAF marker PDGFR-β was minimal in the liver metastases, resulting in liver metastatic tumors with poor stromal components (Figure 2g, 2nd row). Histological analysis revealed no significant differences in the PDGFR-β expression levels or the number of CD8-positive cells in the liver metastatic tumors between the two experimental groups (*p* > 0.05). The Ki67 labeling index was significantly lower in the anti-PD-1 antibody treatment group than that in the control group (*p* < 0.01; Figure 2h). These results suggest that an antitumor effect could be obtained in inflamed CRC liver metastasis using immunotherapy treatment with an anti-PD-1 antibody alone.

### 3.5. Effects of Combined Immunotherapy with Dasatinib on Excluded-Type CRC Liver Metastasis

**Figure 3:**
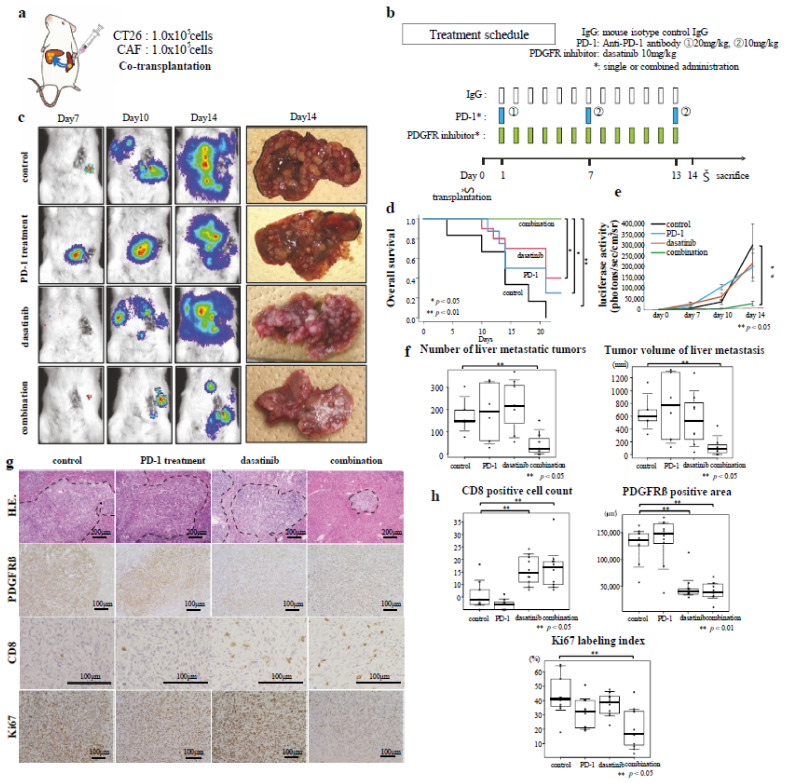
Anti-PD-1 antibody and dasatinib combination treatment and excluded-type colon cancer liver metastasis. The inhibitory effect of combined anti-PD-1 antibody and dasatinib treatment on liver metastasis was evaluated using a mouse model of excluded-type colon cancer. (**a**) Tumor transplantation design and transplanted cell content. (**b**) Treatment schedule (n = 8/group). (**c**) Changes in tumors over time as observed by luminescence imaging (Days 7, 10, and 14 post-transplantation) and gross observation of the tumors after sacrifice on day 14 post-transplantation. (**d**) Kaplan–Meier survival curves: control, n = 6; PD-1, n = 8; dasatinib, n = 10; combination, n = 9. (**e**) Quantification of changes over time according to luciferase activity. (**f**) Tumor number and volume of metastasized tumors. (**g**) Representative micrographs of HE-stained (100× magnification) and immunostained (200× magnification) tumors. Immunostaining was performed using PDGRβ-, CD8-, and Ki-67-specific antibodies. (**h**) Quantification of the number of CD8-positive cells, PDGFRβ-positive area, and Ki67-positive cell percentage of metastatic tumors.

The therapeutic effect of immunotherapy on the excluded-type CRC liver metastases with abundant stromal components was investigated by performing a therapeutic experiment using a liver metastasis mouse model, in which both the CAFs and CT26 cells were co-transplanted into the spleens (Figure 3a,b). A representative image of the liver removed 14 days after transplantation is shown in Figure 3c (right panel). Overall survival was significantly improved in the combined treatment group compared with that in the other experimental groups (*p* < 0.05, *p* < 0.01; Figure 3d). The body weight loss was significantly suppressed in the combined treatment group compared to the other three groups (Supplementary Figure S3B). Luciferase activity was significantly lower in the combined treatment group than in the other groups 14 days post-transplantation (*p* < 0.05; Figure 3e). The number and volume of liver metastatic tumors were significantly lower in the combination treatment group than in the other experimental groups (*p* < 0.05; Figure 3f). The liver metastatic tumors created by co-transplantation exhibited abundant PDGFR-β-positive cells (Figure 3g, 2nd row). Furthermore, it was thought that the co-metastasis of the CAFs with the cancer cells occurred. The number and volume of liver metastatic lesions were higher after co-transplantation with both the CAFs and CT26 cells compared with those of the liver metastatic tumors formed by the single transplantation of the CT26 cells (Figure 3f). Histological analysis revealed that the PDGFR-β expression levels were significantly lower in the dasatinib and combined treatment groups compared to the control and anti-PD-1-antibody only treatment groups (*p* < 0.01; Figure 3h). Furthermore, the number of CD8-positive cells infiltrating the liver metastatic tumor was significantly higher in the dasatinib treatment and combination treatment groups than in the control and anti-PD-1 antibody treatment groups (*p* < 0.05; Figure 3h). The Ki67 labeling index was significantly lower in the combined treatment group than that of the other three experimental groups (Figure 3g,h; *p* < 0.05).

These results suggest that immunotherapy alone was not effective in treating the liver metastatic tumors with abundant stroma, but an antitumor effect was seen with the combined use of immunotherapy and dasatinib. Histological analysis indicated that the addition of the dasatinib to the treatment regimen reduced the amount of stroma, thereby promoting immune cell infiltration into the tumor.

### 3.6. RNA-seq Analysis and GSEA

**Figure 4:**
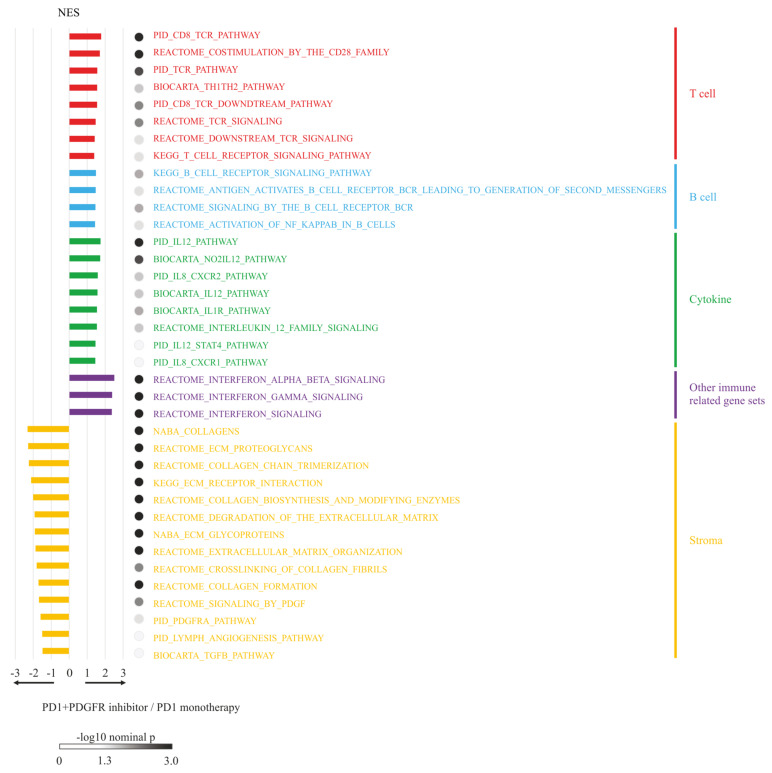
Gene set enrichment analysis (GSEA). RNA-seq was performed to compare the excluded-type liver-metastasis tumors treated with anti-PD-1 antibody monotherapy with those treated with a combination therapy of anti-PD-1 antibody plus dasatinib. Genes are grouped and color-coded according to their major functions. NES, normalized enrichment score. Complete GSEA report data for anti-PD-1 antibody monotherapy and anti-PD-1 antibody plus dasatinib combination therapy are provided in Supplementary Data S1 (Data S1) and Supplementary Data S2 (Data S2).

GSEA revealed that the combined treatment group had multiple enriched immune-related pathways compared to the anti-PD-1 monotherapy group, including T cell-related, B cell-related, and cytokine-related pathways. Interferon gamma- and immune checkpoint-related pathways were also enriched in the combined treatment group. Furthermore, the pathways related to extracellular matrix formation, collagen, and PDGFR signaling were significantly suppressed in the combined treatment group (Figure 4). These results indicate that tumor-related stroma can be suppressed by blocking the PDGFR signaling pathway and promoting immunotherapy-induced T cells.

### 3.7. Flow Cytometry Analysis

**Figure 5:**
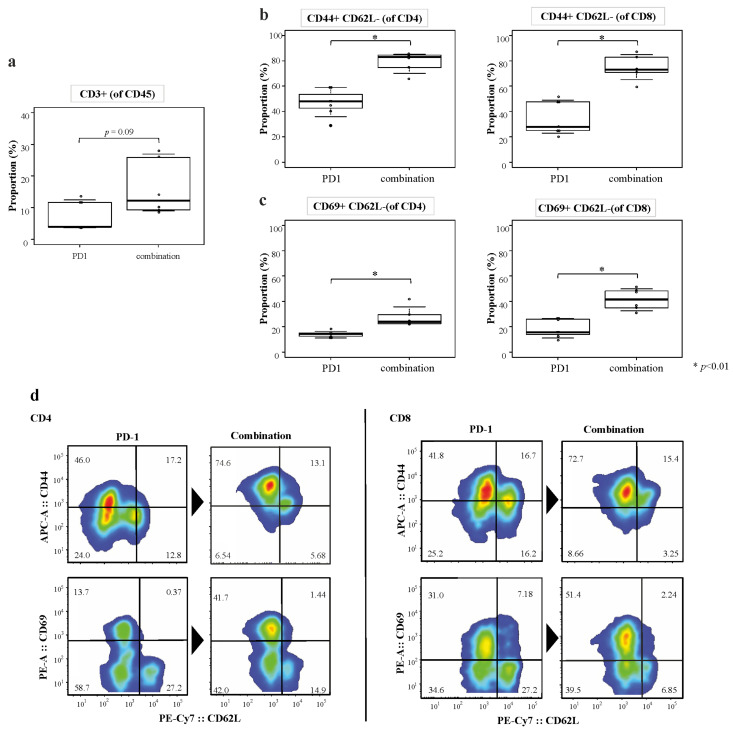
Fluorescence-activated cell sorting (FACS) analysis of liver metastatic tumors. FACS analysis was performed to determine the changes in the tumor immune response associated with the anti-PD-1 antibody alone or combined anti-PD-1 antibody plus dasatinib treatment. (**a**) Quantification of CD3^+^ cells in different experimental groups of mice. (**b**) Quantification of CD4^+^CD44^+^ and CD8^+^CD44^+^ cells in different experimental groups of mice. (**c**) Quantification of CD4^+^CD69^+^ and CD8^+^CD69^+^ cells in different experimental groups of mice. (**d**) Representative FACS graphs of CD4^+^CD44^+^, CD4^+^CD69^+^, CD8^+^CD44^+^, and CD8^+^CD69^+^ cells of mice treated with the anti-PD-1 antibody alone or with a combination of anti-PD-1 antibody and dasatinib.

Flow cytometry analysis revealed that the recruitment of CD3^+^ cells was higher in the combination treatment group than in the anti-PD-1 monotherapy group (Figure 5). Furthermore, the populations of the CD44^+^CD62L^−^ effector memory T cells and CD69^+^CD62L^−^ effector T cells were significantly increased in both the CD4^+^ and CD8^+^ cells in the combined treatment groups (*p* < 0.01; Figure 5). These results suggest that combined therapy promotes the establishment of long-term tumor-specific T-cell responses.

## 4. Discussion

The current study, using clinical specimens, demonstrated an abundant CAF component in the excluded-type CRC tumors, which are a common type of CRC. Furthermore, the number of CAFs in the primary tumor foci correlated with the number of CAFs in the liver metastatic foci. Our findings demonstrate that the administration of dasatinib combined with an anti-PD-1 antibody in an excluded-type CRC liver metastasis mouse model can reduce the amount of stroma in metastatic liver tumors and induce immune cell infiltration, thereby enhancing the therapeutic effect of the immune checkpoint inhibitors.

Immune checkpoint inhibitors are effective in some patients with metastatic CRC that have high microsatellite instability; however, they have limited clinical effects in patients with microsatellite stable metastatic CRC, accounting for approximately 95% of all metastatic CRC cases [7,20,21,22,23]. There are various reports on the mechanisms by which microsatellite stable metastatic CRC remain resistant to immune checkpoint inhibitor monotherapy [24]. CRC is believed to exhibit a small amount of tumor variation, low immunogenicity, and difficulty in activating tumor immunity [25]. Accompanying these characteristics is the exclusion of CD8^+^ T cells from the tumor microenvironment. Multiple reports have indicated that the enrichment of WNT/β-catenin signaling is involved in the elimination of T cells [26,27]. Enriched transforming growth factor beta (TGFβ) signaling is also thought to be an important mechanism of antigenic escape in CRC, and enhanced CAF levels promote fibrosis in tumors and suppress antitumor immunity [28,29]. Furthermore, patients with CRC liver metastasis exhibit reduced T-cell diversity and function in tumors and a low T-cell signature score [30]. These results indicate that liver metastasis may be the cause of resistance to immune checkpoint inhibitors.

CAFs are an important component of the tumor microenvironment, and there is considerable evidence that they play a facilitating role in tumorigenesis [8]. Recent studies have shown that CAFs are associated with the recruitment and regulation of innate and adaptive immune cells [31]. In a mouse model of breast cancer, tumors treated with anti-CAF therapy transitioned from a Th2 to Th1 response with a prominent increase in CD8^+^ T cells and a prominent decrease in tumor-associated macrophages, myeloid-derived suppressor cells, and regulatory T cells [32]. It has also been reported that the enhancement of the TGFβ signal in CAFs is involved in the elimination of T cells from tumors in a urothelial carcinoma mouse model [33]. These findings suggest that CAFs function in a suppressive manner to impede tumor immunity. To overcome this, we used a combination of CAF-targeted therapy and immunotherapy. We hypothesized that this could promote the tumor immune cycle by transforming the TIME into an immune-response phenotype in the presence of effective immune cells. We have previously reported that the administration of dasatinibs imatinib and nilotinib suppresses the growth of CAFs in mouse orthotopically transplanted gastric cancer and CRC tumors [12,19,34]. In the current study, we used dasatinib, which strongly suppresses the PDGFR axis [35]. Our treatment experiment showed that the combined treatment of dasatinib and immunotherapy not only suppresses the stromal reaction and induces immune cell infiltration, but also leads to an increase in CD62L-, CD44+ effector memory cells. This may indicate that the immune response against cancer cells is sustained after treatment and rechallenge experiments could have confirmed this effect.

We found that the co-transplantation of cancer cells and CAFs into the spleens of mice resulted in liver metastatic tumors rich in CAF components. This suggests that CAFs contribute to the formation of metastatic niches and the co-metastasis of cancer cells to the liver. The immunostaining results of the clinical CRC specimens indicated that the primary foci stromal components metastasize to the liver metastatic foci, regardless of the histological phenotype.

Various combinations of drugs such as mitogen-activated protein kinase inhibitors [36,37], cytotoxic anticancer drugs [38,39,40], and anti-cytotoxic T lymphocyte-associated antigen-4 antibodies [7,21] have been reported to improve the efficacy of immunotherapy. Promising results from preclinical and clinical trials have been reported; however, prominent effects have not yet been observed in pancreatic cancer, breast cancer, or CRC. One possible reason for this may be the presence of a large amount of stroma in these tumors. Immunotherapy alters the immune response of patients, but immune system enhancement alone may not be effective in overcoming the immunosuppressive capacity of the tumor microenvironment due to the presence of CAFs.

## 5. Conclusions

We developed an excluded-type CRC liver metastasis model refractory to immunotherapy alone. However, the combined use of immunotherapy and dasatinib, which specifically targets CAF-associated stroma, alleviates the immunosuppressive microenvironment, and activates immune cells that exert antitumor effects. This combined therapy may be effective in treating patients with metastatic CRC for whom immunotherapy alone does not provide a satisfactory outcome.

## Data Availability

The datasets used and/or analyzed during the current study are available from the corresponding author upon reasonable request.

## Supplementary Materials

The following supporting information can be downloaded from: https://www.mdpi.com/article/10.3390/cancers14246146/s1, Figure S1: Tumor immune microenvironment (TIME) classification of human colorectal cancer (CRC) specimens using CD8 immunostaining; Figure S2: Effect of dasatinib on cancer-associated fibroblasts (CAF) and CT26 cell proliferation; Figure S3: Body weight chart of mice in each treatment and control group; Table S1: Clinicopathological features and phenotype classification of study participants and resected col-orectal cancer (CRC) specimens (n = 165); File S1 (.docx): Supplementary Text, Supplementary Materials, and Methods; Data S1 (.xlsx): Monotherapy gene set enrichment analysis report; Data S2 (.xlsx): Gene set enrichment analysis of combination therapy.
