# Supplementary material for: Anti-Programmed Cell Death-1 Antibody and Dasatinib Combination Therapy Exhibits Efficacy in Metastatic Colorectal Cancer Mouse Models
Source: Cancers (Basel). 2022 Dec 13;14(24):6146. doi: 10.3390/cancers14246146 (PMC9776338; doi:10.3390/cancers14246146)
Supplement: Supplementary file 1 [file cancers-14-06146-s001.zip › File S1.pdf]

Supplementary Text

Supplementary Materials and Methods

Primary antibodies

The primary antibodies included monoclonal rabbit anti-CD8 (Abcam, Cambridge, UK), monoclonal rabbit anti-CD8 (Cell Signaling Technology, Danvers, MA, USA), polyclonal rabbit anti-phosphorylated PDGFR-β (p-PDGFR-β; Santa Cruz Biotechnology, Santa Cruz, CA, USA), rabbit anti-α-smooth muscle actin (α-SMA; Abcam), Ki-67-equivalent (MIB-1; Dako, Carpinteria, CA, USA), and mouse IgG isotype control (Bio X Cell) antibodies. The antibodies used for fluorescence-activated cell sorting (FACS) included PE anti-mouse CD326 (epithelial cellular adhesion molecule: EpCAM; cloneG8.8; BioLegend, San Diego, CA, USA), PE anti-mouse CD140a (PDGFR- α ; clone G8.8; BioLegend), PerCP/Cyanine5.5 anti-mouse CD45 (clone 30-F11; BioLegend), Brilliant Violet 421 anti-mouse CD3ε (clone 145-2C11; BioLegend), PE/Cyanine7 anti-mouse CD4 (clone GK1.5; BioLegend), APC/Cyanine7 anti-mouse CD8a (clone 53-6.7; BioLegend), APC anti-mouse/human CD44 (clone IM7; BioLegend), PE/Cyanine7 anti-mouse CD62L (clone MEL-14; BioLegend), and PE anti-mouse CD69 (clone H1.2F3; BioLegend) antibodies.

Classification of the tumor immune microenvironment (TIME) of human CRC specimens and evaluation of stroma amounts

The TIME of human CRC specimens into immune-tissue phenotypes of inflamed, excluded, or desert types based on CD8 immunostaining. The inflamed type exhibited CD8-positive cells that infiltrated the cancer cell foci and were in direct contact with cancer cells (Supplementary Fig. S1a, Additional File 2). The excluded type exhibited CD8-positive cells in the stroma adjacent to

the cancer cell foci but failed to have direct contact with the cancer cells (Supplementary Fig. S1b, Additional File 2). The desert type demonstrated less than ten CD8-positive cells in five randomly selected microscopic fields at 200x magnification (Supplementary Fig. S1c, Additional File 2). Stroma amounts were determined using α-SMA immunostaining with the proportion of α-SMA-positive areas being evaluated in the primary and liver-metastasis CRC foci. The correlation of the amount of α-SMA-positive area in the primary and liver-metastasis foci was also determined for cases of liver metastasis. Observations were made using a BZ-X710 all-in-one fluorescence microscope (KEYENCE, Osaka, Japan). Five different microscopic fields that included the foci were photographed at 200× magnification for each specimen and analyzed. All the micrographs were obtained under the same conditions (exposure time, gain, illumination light intensity, and aperture stop). The α-SMA-positive areas were determined by arranging and quantifying the brightness thresholds using the BZ-H3C hybrid cell count application of the BZ-X analysis software, version 1.3.1.1 (KEYENCE) (Figure 1a, 1b, 1c). Analyses were performed considering the average value of five microscopic fields of each sample as a single sample.

Cancer-associated fibroblast (CAF) preparation

To generate CAFS, 50 μL HBSS containing 1×10^5 CT26 cells was injected subcutaneously into BALB/c mice. After 14 d, the engrafted tumors and surrounding tissues were collected, and the tumor specimens were shredded using a scalpel. The shredded tissue was incubated with the dispase solution at 37°C for 60 min. The cells were isolated by pipetting and cultured in DMEM supplemented with 10% FBS and penicillin-streptomycin for 24–48 h. The cultured cells were treated with 0.05% trypsin for one minute, and floating cells were collected and seeded into new culture dishes. This method was repeated every three days for a total of three subcultures to

facilitate the isolation of fibroblast-like spindle-shaped cells. The processed cells were sorted by FACS to isolate $CD45^{-}EpCAM^{-}PDGFR^{+}$ cells which were used as CAFs in subsequent experiments.

Inflamed- and excluded-type CRC liver-metastasis mouse models

HBSS (50 µL) containing 1×10^5 CT26 cells was injected into the spleens of female BALB/c mice using a zoom stereomicroscope (Carl Zeiss, Oberkochen, Germany) to create liver metastatic tumors with few stromal components. This was used as an inflamed-type CRC liver-metastasis mouse model. Similarly, 50 µL HBSS containing 1×10^5 CT26 cells and 1×10^5 CAFs was injected into female BALB/c mice to create liver metastatic tumors with rich stromal components. This was used as an excluded-type CRC liver-metastasis mouse model. Luciferase-expressing CT 26 cells were used to track changes in the transplanted tumors over time using a Lumina II *in vivo* imaging system (Xenogen, Alameda, CA, USA).

Therapeutic experiments using CRC liver-metastasis mouse models

To evaluate the efficacy of the anti-PD-1 antibody treatment, an inflamed-type CRC liver-metastasis model was established, and the animals were divided into treatment and control groups. For the anti-PD-1 antibody treatment group, mouse anti-PD-1 antibody was intraperitoneally administered at 20 mg/kg on day 1 post-transplantation of CT26 cells and at 10 mg/kg on days 7 and 13 post-transplantation for a total of three treatments. The control group received 200 µL isotype control IgG antibody (10 mg/kg) on each treatment day. To track the changes over time, the tumors were imaged *in vivo* on days 7, 10, and 14 post-transplantation

using luminescence imaging. The mice were sacrificed on day 14 post-transplantation, and tissue specimens were collected during necropsy, as described below.

To evaluate the efficacy of the combination treatment, an excluded-type CRC liver-metastasis model was established, and the animals were divided into four treatment groups: anti-PD-1-antibody treatment, dasatinib treatment, combined treatment, and control groups. The anti-PD1-antibody treatment group was intraperitoneally administered 20 mg/kg mouse anti-PD-1 antibody 1 d post-transplantation and 10 mg/kg on days 7 and 13 post-transplantation for a total of three treatments. The dasatinib treatment group received daily oral administration of 10 mg/kg dasatinib on days 1–13 post-transplantation. The combined treatment group was intraperitoneally administered 20 mg/kg mouse anti-PD-1 antibody on day 1 post-transplantation and 10 mg/kg on days 7 and 13 post-transplantation, in addition to the daily oral administration of 10 mg/kg dasatinib on days 1 to 13 post-transplantation. The control group was intraperitoneally administered 10 mg/kg isotype control IgG on days 1, 7, and 13 post-transplantation along with daily oral administration of 10 mg/kg isotype control IgG. The tumors were imaged *in vivo* on days 7, 10, and 14 post-transplantation using a Lumina II system. The mice were monitored until they died due to natural causes or until they were sacrificed on day 14 post-transplantation. Tissue specimens were collected during the necropsy, as described below.

Necropsy and histological evaluation

The mice were sacrificed by cervical dislocation under anesthesia, and the tumor volumes and the number of liver metastatic tumors were recorded. The shole liver that was removed was sliced at 2 mm intervals, and the area of the metastatic tumor found in both lateral cross-sections was calculated as πr^2, and the volume of each metastasis was calculated assuming a depth of 1

mm. They were summed to obtain the tumor volume. Tumor tissue specimens were fixed in formalin, embedded in paraffin, and serially sectioned at 4-µm thickness. The tissue sections were immunostained with PDGRβ-, CD8-, and Ki-67-specific antibodies. All specimens were examined and photographed using a BZ-X710 all-in-one fluorescence microscope, as described above. BZ-H3C hybrid cell count and BZ-H3CM microcell counting applications of BZ-X analysis software were used to quantify the PDGFR-β-positive areas, number of CD8-positive cells, and proportion of Ki67-positive cells.

RNA-sequencing (RNA-seq) analysis and gene set enrichment analysis (GSEA)

Excluded-type CRC liver metastatic tumors from mice treated with anti-PD-1 antibodies as monotherapy and combined therapy with dasatinib were dissected and mechanically disrupted using a homogenizer. Total RNA was extracted from tissue homogenates using the Qiagen RNeasy Mini Kit, according to the manufacturer's protocol. Library construction and data processing were performed at the Beijing Genome Institute (Beijing, China). The library was sequenced using the DNBSEQ-G400RS platform and high-quality reads were obtained. Sequence alignment was conducted against the GRCm38 mouse reference genome version GCF_000001635.26_GRCm38.p6 (https://www.ncbi.nlm.nih.gov/assembly/GCF_000001635.26).

The RNA-seq data is registered with GEO and the accession number is GSE218603. (https://www.ncbi.nlm.nih.gov/geo/query/acc.cgi?acc=GSE218603)

Human sequence data were downloaded from Mouse Genome Informatics (http://www.informatics.jax.org/) and used to annotate mouse genes with human genes. After

genes with reads per kilobase of transcript per million reads mapped = 0 were removed, the samples were analyzed via GSEA to evaluate the differential modulation of molecular pathways.

FACS analysis of immune-cell surface antigen expression

As noted, mice with established excluded-type CRC liver metastasis were used for the therapeutic mouse model. The mice were divided into two experimental groups: one treated with anti-PD-1 antibody alone and the other treated with anti-PD-1 antibody in combination with dasatinib. Necropsy was performed on day 14 post-transplantation and the liver metastatic tumors were removed. BD Horizon Dri Tissue & Tumor Dissociation Reagent (Becton, Dickinson and Company, USA) was used to disrupt the tumors into single-cell suspensions, according to the manufacturer's protocol. Individual cells were immunostained using the indicated antibodies, and the expression levels of immune cell surface antigens were evaluated using FACS. Flow cytometry was performed using LSRFortessa X-20 (Becton Dickinson).
