# Supplementary material for: Anti-Programmed Cell Death-1 Antibody and Dasatinib Combination Therapy Exhibits Efficacy in Metastatic Colorectal Cancer Mouse Models
Source: Cancers (Basel). 2022 Dec 13;14(24):6146. doi: 10.3390/cancers14246146 (PMC9776338; doi:10.3390/cancers14246146)
Supplement: Supplementary file 1 [file cancers-14-06146-s001.zip › Supplemnetary figures and tables.pdf]

Supplementary Figures and Tables:
Anti-Programmed Cell Death-1 Antibody and Dasatinib Combination Therapy Exhibits Efficacy in Metastatic Colorectal Cancer Mouse Models

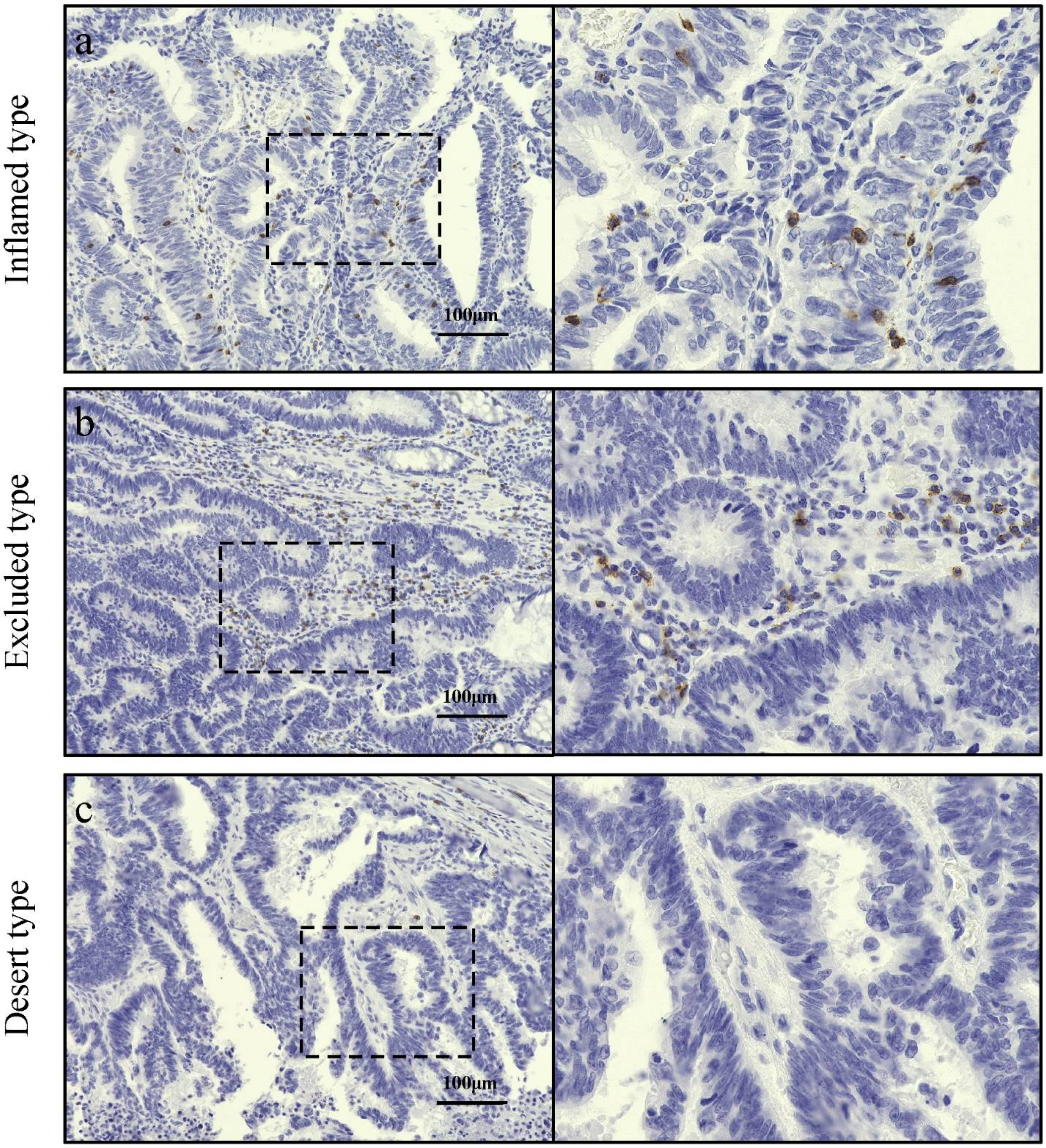

Figure S1. Tumor immune microenvironment (TIME) classification of human colorectal cancer (CRC) specimens using CD8 immunostaining. The CRC specimens were classified into immune-tissue phenotypes. (**A**) Inflamed type CRC specimens exhibit CD8-positive cells infiltrating the cancer cell foci and are in direct contact with the cancer cells. (**B**) Excluded type CRC specimens exhibit CD8-positive cells in the stroma adjacent to the cancer cell foci. No

direct contact of CD8-positive cells with the cancer cells are observed. (**C**) Desert type CRC specimens demonstrate a very limited number of CD8-positive cells. The left column of micrographs had a 200× original magnification. The size bars represent 100 μm. The right column of micrographs are enlargements of the regions indicated by the dashed-line boxes of the left column micrographs. The immunostained CRC specimens were counterstained with hematoxylin.

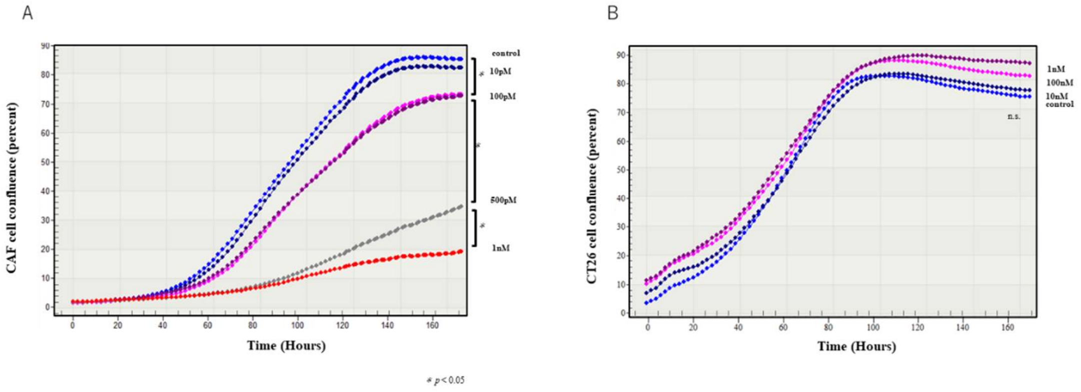

Figure S2. Effect of the PDGFR inhibitor dasatinib on cancer-associated fibroblast (CAF) and CT26 cell proliferation. (**A**) Dasatinib suppresses CAF proliferation in a concentration-dependent manner. (**B**) Dasatinib fails to suppress CT26 proliferation.

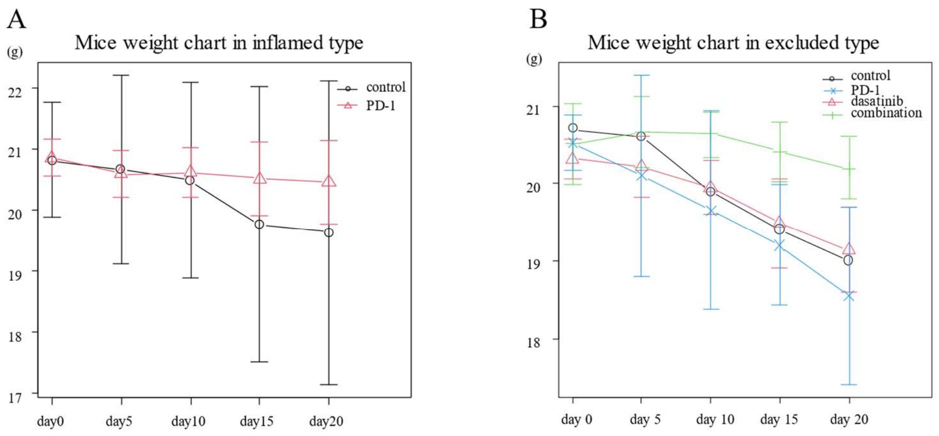

Figure S3. Body weight chart of mice in each treatment and control group. (**A**) Weight change of anti-PD-1 antibody treated group and control group in inflamed type. (**B**) Weight change of each treatment and control group in excluded type.

Table S1. Clinicopathological features and phenotype classification of study participants and resected colorectal cancer (CRC) specimens (n = 165).

Variable		Phenotype Classification<br>Inflamed-Type<br>n = 17 (10.3%)	Excluded-Type<br>n = 90 (54.5%)	Desert-Type<br>n = 58 (35.2%)	*p*-value
Age (years)		72.2 ± 10.2	67.4 ± 10.2	65.2 ± 13.9	0.086
Sex	Male	10 (59%)	57 (63%)	32 (55%)	0.61
	Female	7 (41%)	33 (37%)	26 (45%)	
Location	Colon	13 (76%)	67 (74%)	47 (81%)	0.713
	Rectum	4 (24%)	23 (26%)	11 (19%)	
T Classification	T1,2	6 (35%)	24 (27%)	18 (31%)	0.712

	T3,4	11 (65%)	66 (73%)	40 (69%)	
N Classification	N0	11 (65%)	46 (51%)	29 (50%)	0.543
	N1–3	6 (35%)	44 (49%)	29 (50%)	
M Classification	M0	14 (82%)	72 (80%)	41 (71%)	0.362
	M1	3 (18%)	18 (20%)	17 (29%)	
pStage	Stage I,II	11 (65%)	40 (44%)	25 (43%)	0.262
	Stage III,IV	6 (35%)	50 (56%)	33 (57%)	
Lymphatic Invasion	Ly0,1	17 (100%)	74 (82%)	46 (79%)	0.13
	Ly2,3	0 (0%)	16 (18%)	12 (21%)	
Venous Invasion	V0,1	17 (100%)	76 (84%)	48 (83%)	0.191
	V2,3	0 (0%)	14 (16%)	10 (17%)	
Histological Type	tub1, tub2, pap	15 (88%)	81 (90%)	54 (93%)	0.75
	por, muc	2 (12%)	9 (10%)	4 (7%)	

Abbreviations: tub1, well differentiated adenocarcinoma; tub2, moderately differentiated adenocarcinoma; por, poorly differentiated adenocarcinoma; muc, mucinous carcinoma.
